# Dynamic Interfacial Tensions of Surfactant and Polymer Solutions Related to High-Temperature and High-Salinity Reservoir

**DOI:** 10.3390/molecules28031279

**Published:** 2023-01-28

**Authors:** Xiang-Long Cui, Yi Pan, Fu-Tang Hu, Lu Han, Xiu-Yu Zhu, Lei Zhang, Zhao-Hui Zhou, Gen Li, Gui-Yang Ma, Lu Zhang

**Affiliations:** 1 College of Petroleum Engineering, Liaoning Petrochemical University, Fushun 113001, China; 2 Research Institute of Drilling and Production Technology, PetroChina Qinghai Oilfield Company, Dunhuang 736202, China; 3 State Key Laboratory of Enhanced Oil Recovery (PetroChina Research Institute of Petroleum Exploration & Development), Beijing 100083, China; 4 Key Laboratory of Photochemical Conversion and Optoelectronic Materials, Technical Institute of Physics and Chemistry, Chinese Academy of Sciences, Beijing 100190, China; 5 Department of Petroleum Engineering, Northeast Petroleum University, Daqing 163318, China

**Keywords:** betaine, polymer, interfacial tension, n-alkanes, crude oil

## Abstract

Betaine is a new surfactant with good application prospects in high-temperature and high-salinity reservoirs. The interfacial properties of two kinds of betaine mixtures with a good synergistic effect were evaluated in this paper. On this basis, the effects of temperature-resistant, salt-resistant polymers with different contents of 2-acrylamide-2-methylpropanesulfonic acid (AMPS) on dynamic interfacial tensions (IFTs) against n-alkanes and crude oil were studied. The experimental results show that the IFTs between betaine ASB and n-alkanes can be reduced to ultra-low values by compounding with anionic surfactant petroleum sulfonate (PS) and extended anionic surfactant alkoxyethylene carboxylate (AEC), respectively. ASB@AEC is very oil-soluble with n_min_ value ≥14, and ASB@PS is relatively water-soluble with n_min_ value of 10. The water solubility of both ASB@PS and ASB@AEC is enhanced by the addition of water-soluble polymers. The HLB of the ASB@AEC solution becomes better against crude oil after the addition of polymers, and the IFT decreases to an ultra-low value as a result. On the contrary, the antagonistic effect in reducing the IFT can be observed for ASB@PS in the same case. In a word, polymers affect the IFTs of surfactant solutions by regulating the HLB.

## 1. Introduction

As oil extraction continues, conventional reservoir development can no longer meet the growing oil demand, and the focus of development has begun to shift to high-temperature, high-salt, low-permeability, non-homogeneous, and severe secondary and tertiary reservoirs. Due to complex reservoir conditions, a significant amount of residual crude oil remains in the reservoir after primary and secondary oil recovery [1,2]. In order to improve the recovery rate, chemical oil drive has become the main method of tertiary oil recovery [3].

Chemical flooding includes alkali flooding, surfactant flooding, and polymer flooding, as well as their binary and ternary combinations, such as alkali–surfactant (AS) flooding, surfactant–polymer (SP) flooding, and alkali–surfactant–polymer (ASP) flooding [4,5,6]. In AS systems, the ability of flooding to reduce the oil–water interfacial tension (IFT) is higher than that of alkali or surfactant alone, but the sweep area cannot be expanded [7]. However, in the ASP system, surfactants mainly reduce the IFT between oil and water to improve the efficiency of oil displacement. On the other hand, polymers can increase the viscosity of the oil displacement system, reduce the water-to-oil ratio, and improve sweep efficiency [8,9]. With the wide application of ASP technology, a series of problems also appear [10,11]. The common use of alkali reduced permeability and corrosion equipment, etc. In addition, the SP system not only has the advantages of ASP, but also receives more and more attention because of its low operating costs, wide application in oilfields, and environmental protection [12,13].

Currently, the common surfactants for enhanced oil recovery (EOR) are mainly anionic or anionic and ionic mixed surfactants. When anionic surfactants encounter divalent inorganic salt ions, a salt precipitation effect occurs and thus they lose their activity; meanwhile, non-ionic surfactants have poor temperature resistance due to their turbidity points, so conventional surfactants for EOR can no longer meet the needs of high-temperature and high-salinity reservoirs. Compared with conventional surfactants, amphoteric surfactants have a special molecular structure: the hydrophilic part can contain both anions and cations and exist as amphoteric ions in a wide pH range, and are less affected by temperature and mineralization, and have good resistance to divalent ions [14,15]. Betaine surfactant is one of the most important amphoteric surfactants with good interfacial properties and can achieve an ultra-low IFT with crude oil under suitable conditions [16]. The electrically neutral betaine molecule has almost no effect on the viscosity of the polymer solution and is low in toxicity and biodegradable, which meets environmental requirements and has good prospects for application in high-temperature and high-salt reservoir environments [17,18].

It is well known that a single surfactant is difficult to cope with the complex reservoir environment. Zhou et al. demonstrated that it is difficult for a betaine molecule with a large difference between the sizes of hydrophilic and hydrophobic groups to produce an ultra-low IFT by itself [19]. Cao et al. studied the IFTs of betaines with different sizes of hydrophobic parts against hydrocarbons and found the values of IFTs decrease with an increase in the size of the hydrophobic part, but still could not reach an ultra-low level [20]. Therefore, it is significant to study the synergistic effect between betaine and other types of surfactants to improve the interfacial properties. Sun et al. found that betaine ASB has a strong synergistic effect with petroleum sulfonate and can achieve an ultra-low IFT in a wide concentration range [21]. Li et al. used a mixture of alkylated diphenylmethane sulfonate and didodecyl methyl carboxyl betaine to reduce the IFT between Shengli crude oil and formation water to 5 × 10^−3^ mN/m at 70 °C [22]. Zhong et al. found that when betaine ACB was mixed with anionic-nonionic surfactant C_18_E_3_C. The positive group of the ACB molecule and the negative group of the C_18_E_3_C molecule attracted each other through electrostatic attraction and formed a compact mixed adsorption film with crude oil active substance, which made the IFT reach an ultra-low level [23]. Zhong et al. also found a strong synergistic effect between ASB, ionic surfactants, and natural surfactant to produce ultra-low IFT for crude oil over a wider concentration range [24]. Zhou et al. found that when combined with the anionic surfactant sodium dodecyl sulfate (SDS), the base could react with petroleum acid to produce in situ surfactant under alkaline conditions, which could be combined with the EAPB-SDS combination system to achieve the ultra-low IFT [25]. The above experimental results show that there is a strong synergistic effect between betaine and anionic-nonionic or ionic surfactants, which further reduce the IFT. 

In the middle and late stages of oil field development, chemical flooding compound surfactant and surfactant is gaining more and more attention in order to extend the field development life and increase the recovery rate [26]. Ma et al. studied the effect of partially hydrolyzed polyacrylamide (HPAM) on the IFT between anionic and negative non-ionic surfactants and found that the polymer and surfactant interact to form a mixed adsorption film [27]. The concentration of HPAM will affect the tightness of the interfacial membrane structure and the IFT. Li et al. studied the dynamic IFTs between surfactant/polymer/organic base system and homolog series alkanes and found that HPAM could significantly improve the water solubility of surfactant and organic base solution, and reduce the IFT against low alkyl carbon number hydrocarbons [28]. SiTu et al. found that the addition of polymers changed the arrangement of surfactant molecules at the interface, causing a significant impact on the dynamic IFTs behavior and equilibrium values between betaine solution, and n-alkanes [29]. Li et al. studied the effects of HPAM and hydrophobically modified polyacrylamide (HMPAM) on the IFT between the betaine (ASB, BSB) and decane. The HPAM could prevent hydrophilic groups in ASB molecules from lying flat and produce the low IFT, while HMPAM and ASB molecules formed mixed aggregate at the interface, which destroyed the arrangement of ASB molecules, and led to the increase in IFT. Due to the tight interfacial membrane formed by BSB molecules and the existence of steric hindrance, the addition of two polymers has almost no effect on IFT in the BSB system [30]. 

In recent years, with the continuous development of oil fields, high-temperature and high-mineralization reservoirs have gradually become the focus of development. Conventional HPAMs are prone to hydrolyze under high-temperature and high-salt conditions to produce high concentrations of carboxylic acids and complex with calcium and magnesium ions to produce precipitation, resulting in a significant reduction in polymer viscosity, stability, and oil drive performance [31,32]. Therefore, how to inhibit the polymer hydrolysis becomes the key to solve the chemical drive in ultra-high-temperature reservoirs. At present, the hydrolysis of HPAM is primarily inhibited by the introduction of the monomeric 2-acrylamide-2-methylpropanesulfonic acid (AMPS) [33,34,35]. Although a large number of studies have shown that the introduction of AMPS monomer increases the polymer viscosity and provides good stability and performance, which in turn improves the recovery, little has been reported on how it affects the IFT of betaine and anionic and anionic–nonionic complex systems. In this paper, the effect of polymers with different AMPS monomer contents on the IFT of betaine complexing (ASB@AEC, ASB@PS) solutions against n-alkanes and Shengli crude oil are investigated. The related results have important guiding significance for the design of enhanced recovery formulations in high-temperature and high-salt reservoirs.

## 2. Results and Discussion

### 2.1. Interfacial Tension between Surfactant Solution and n-Alkanes

#### 2.1.1. Effect of Concentration

The application of surfactants in recovery enhancement is mainly based on their ability to reduce the IFT value between oil and water. When surfactant molecules adsorb at the interface, the difference between oil and water is diminished. The interaction of hydrophobic groups of surfactants far exceeds the original interaction between oil and water molecules, and at the same time, the hydrophobic fraction is similar in size to achieve ultra-low IFT. To evaluate the difference in interfacial activity of ASB@AEC and ASB@PS, the dynamic IFTs between betaine solutions with different concentrations and n-decane were measured.

**Figure 1:**
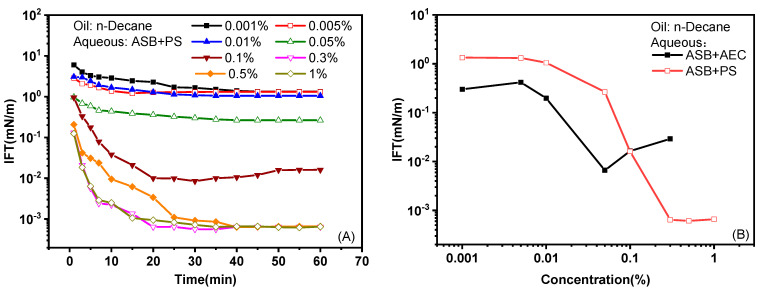
Effect of concentration on dynamic IFTs of ASB@PS (**A**) and equilibrium IFTs of ASB@PS and ASB@AEC (**B**) solutions against n-decane.

As can be seen from Figure 1A, the dynamic IFT decreases continuously with time, and the dynamic curve has a typical “L” shape [36]. This dynamic process indicates that the surfactant molecules are continuously aggregating at the interface, and when the surfactant molecules almost completely replace the solvent molecules at the interface, an ultra-low IFT can be achieved. As can be seen from Figure 1B, with the increase in ASB@PS concentration, the equilibrium IFT value gradually decreases to the order of 10^-4^ mN/m. When the concentration is 0.3 wt%, the steady state value of IFT will not decrease. For the ASB@PS solution, the PS molecule increases the size of the hydrophobic part of the mixed system, which is similar to the size of the hydrophilic group. Moreover, PS molecule and betaine ASB molecule are attracted by electrostatic interaction, which leads to the close arrangement of the adsorbed molecules at the interface and achieves an ultra-low IFT, which indicates that ASB@PS has strong interfacial activity. This is consistent with the finding by Zhong et al. that zwitterionic and anionic surfactants exert synergistic effects on the generation of ultra-low IFTs over a wide range of concentrations [24]. It is worth noting that an ultra-low IFT can be achieved at 0.05 wt% for the ASB@AEC solution, but only reaches the order of 10^−2^ mN/m at 0.3 wt%, which is much higher than the IFT between ASB@PS and n-decane. This may be due to the larger EO size of AEC, which needs an optimized ratio of ASB and AEC molecules at the interface. Moreover, the higher IFT value of ASB@AEC in comparison with ASB@PS can be logically explained by the difference in hydrophilic–lipophilic balance (HLB), which will be discussed later.

#### 2.1.2. Effect of Carbon Chain Length of Oil Molecule

The dynamic IFTs between 0.3 wt% surfactant solutions and n-alkanes were tested in order to further understand the mechanism leading to the reduction in the oil–water IFT. As we all know, the HLB of surfactant is an important factor to determine its ability to reduce IFT, which can be measured by n-alkane scanning of IFT. At a certain temperature and salinity, the IFT of a fixed concentration of surfactant solution will pass through a minimum value when scanning the n-alkanes. This alkane carbon number is called the n_min_ value of the surfactant [37]. The n_min_ value can quantitatively characterize the HLB of surfactants: the higher the n_min_ value, the stronger the oil-solubility, and vice versa [38].

**Figure 2:**
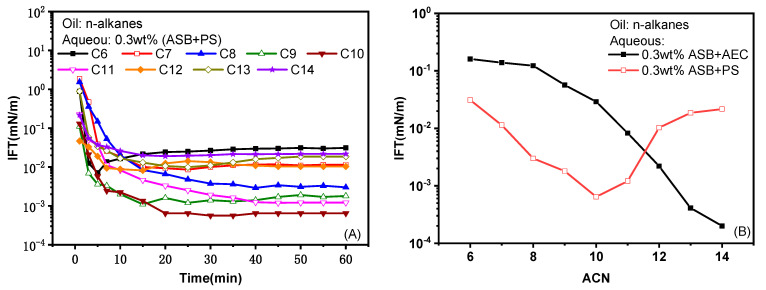
Dynamic IFTs of 0.3 wt% ASB@PS solution (**A**) and equilibrium IFTs of 0.3 wt% ASB@AEC and ASB@PS solutions (**B**) against n-alkanes.

From Figure 2A, it is clear that the dynamic IFT behaviors of ASB@AEC and ASB@PS are similar to that in Figure 1A, showing a typical “L” curve. The equilibrium IFT values of ASB@AEC pass through a minimum with the increase in the alkane carbon number and the n_min_ value ≥14, indicating that it is very oil-soluble. The n_min_ value of ASB@PS is 10, which indicates that it is relatively water-soluble. Both of these systems can achieve the ultra-low IFT with n-alkanes with appropriate chain length. As we mentioned above, when the oil is n-decane, ASB@PS shows the best HLB, the IFT value can be reduced to ultra-low, and ASB@AEC only reduce to 10^−2^ mN/m. However, for the oil C12–C14, ASB@AEC has a better HLB and produces ultra-low IFT values, but the ASB@PS only reduces to 10^−2^ mN/m.

#### 2.1.3. Effects of Polymers

The main type of flooding polymer is water-soluble polyacrylamide, which has a great influence on the solubility of surfactant in water, and then affects the adsorption of surfactant molecules on the oil–water interface. By scanning the IFT between the polymer–surfactant system and the n-alkanes, the influence of the polymer on the HLB of the surfactant can be quantitatively reflected.

**Figure 3:**
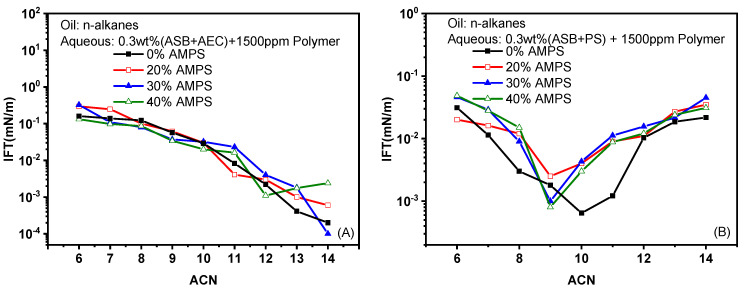
Effect of polymers on equilibrium IFT between 0.3 wt% ASB@AEC (**A**) and ASB@PS (**B**) solutions against n-alkanes.

In order to understand the effects of polymers with different AMPS monomer contents on the HLB of different betaine complex systems, the effects of three polymers with a concentration of 1500 ppm on the IFTs between 0.3 wt% ASB@AEC and ASB@PS solutions and n-alkanes were tested. The monomer contents of the AMPS are 20 wt%, 30 wt%, and 40 wt%, referred to as 1#, 2#, and 3#, respectively. As shown in Figure 3A, the AMPS monomer content in the polymer below 40 wt% has no effect on the n_min_ values of the ASB@AEC system. Adding polymer 3# reduces the n_min_ of the ASB@AEC system to 12 and slightly increases its water solubility, reflecting the strong hydrophilicity of polymer on the HLB of ASB@AEC. The higher the AMPS content of polymer molecules, the greater the impact on the HLB of surfactants. Figure 3B, the presence of monomer AMPS also enhances hydrophilicity, which further affects the HLB of ASB@PS, reducing its n_min_ value to nine.

The effect of polymer on the dynamic IFTs curve of different complex systems is different, which may show an “L” shape when decreasing to the plateau value and a “V” shape when passing the lowest value with time [39,40,41]. In order to further understand the effect of AMPS monomer polymer on the dynamic IFTs of surfactant solution, the dynamic IFTs between polymer with different concentrations and 0.3 wt% ASB@AEC/ASB@PS solutions against n-decane were studied.

**Figure 4:**
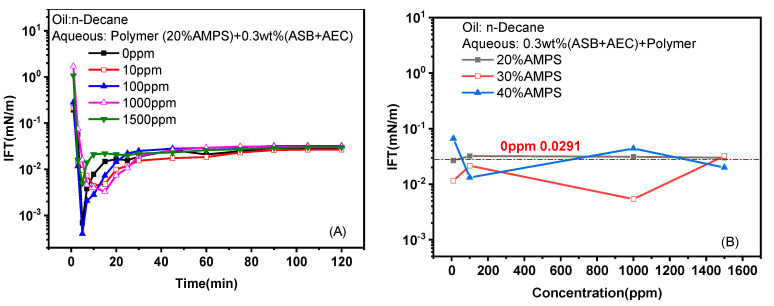
Effect of polymer concentration on dynamic (**A**) and equilibrium (**B**) IFTs between 0.3 wt% ASB@AEC solution and n-decane.

As can be seen from Figure 4A, whether polymer is added or not, the dynamic IFT curves of ASB@AEC show a “V” shape. The instantaneous IFT can reach an ultra-low level in a short time, indicating that the mixture adsorption of ASB and AEC molecules on the interface can form a tight interfacial membrane in a short time and reduce the IFT to an ultra-low level. With the change in time, the orientation of ASB@AEC molecules on the interface changes, the hydrophilic groups are tiled to the interface, the space occupied by molecules becomes larger, and some molecules are desorbed from the interface, leading to the increase in the value of dynamic IFTs [38]. As can be seen from Figure 4B, adding different concentrations of polymer, the overall IFT decreased; at the best conditions, the IFT value can be ultra-low. The n_min_ value of ASB@AEC is 14, and the surfactant molecules tend to be distributed in the oil phase when the oil phase is n-decane. The addition of polymer increases the water solubility of the surfactant, improves the HLB of the original system, and increases the number of ASB@AEC molecules on the interface, resulting in a decrease in the IFT.

**Figure 5:**
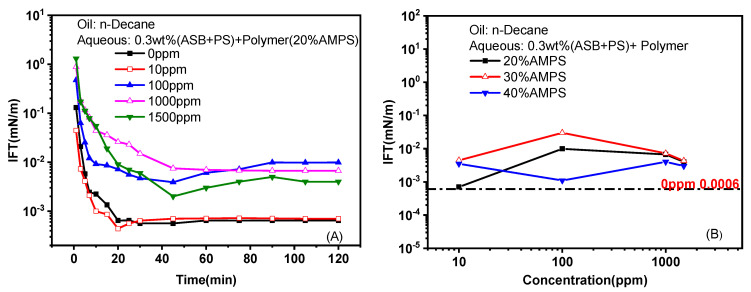
Effect of polymer concentration on dynamic (**A**) and equilibrium (**B**) IFTs between 0.3 wt% ASB@PS solution and n-decane.

From Figure 5A, the dynamic IFT curves of ASB@PS show an “L” shape, this may indicate that the “V” shape of ASB@AEC mainly comes from the arrangement of the EO group at the interface. The addition of polymers has no effect on the trend of dynamic IFTs, but different concentrations of polymers prolong the time taken to reach an equilibrium IFT in the ASB@PS system to different degrees. This is mainly because the polymer can increase the viscosity of the surfactant solution. The higher the concentration, the greater the viscosity of the system will be, which will hinder the transfer of substances and the adsorption of the interface and prolong the time taken for the surfactant to reach the interface. From Figure 5B, the IFT between 0.3 wt% ASB@PS and n-decane increases clearly when different concentrations of polymers are added; this is completely different from that of the ASB@AEC system. This is mainly because the n_min_ value of ASB@PS is 10 and the oil phase is n-decane (the best HLB condition). The addition of polymers increases the water solubility of the surfactant, breaks the HLB, and reduces the interfacial concentration of the ASB@PS molecules, resulting in an increase in the IFT.

In general, the polymer molecules influence the IFTs of surfactant solutions mainly in the following ways: competitive adsorption, mixed adsorption, formation of interfacial aggregates, and changes in hydrophilic–lipophilic balance. Due to the strong interfacial activity of the surfactant studied in this paper, it is difficult for macromolecular polymers to adsorb directly into the oil–water interface, and competitive adsorption or mixed adsorption with surfactant molecules occurs rarely. At the same time, due to the lack of hydrophobic blocks in polymer molecules, the ability to form interfacial aggregates is not strong. Therefore, the polymer mainly changes the solubility of the surfactant and affects the IFT by regulating the hydrophilic–lipophilic balance.

### 2.2. IFT between Surfactant Solution and Shengli Crude Oil

Crude oil contains very complex substances, among which many naturally active substances may act synergistically with surfactants to reduce the IFT between surfactants and crude oil [42,43]. Petroleum acids, such as asphaltenes or naphthenic acids, adsorb to the oil–water interface and solid–oil interface, and affect the interfacial tension, emulsification, and three-phase contact angle of chemical flooding [44,45,46,47]. To further investigate the synergistic effect in the two mixed systems, ASB@AEC and ASB@PS, we studied the IFTs between the mixed systems and crude oil. In this work, the dynamic IFT behaviors of all the mixed solutions against crude oil are similar to those against decane. For brevity, no dynamic data plots are listed.

**Figure 6:**
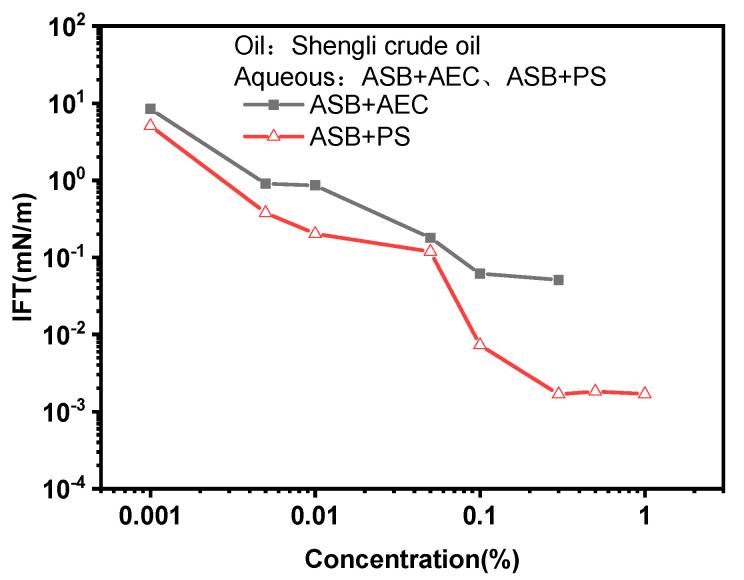
Effect of concentration on equilibrium IFTs of ASB@PS and ASB@AEC solutions against Shengli crude oil.

One can see from Figure 6 that the IFTs decrease with an increase in bulk concentration of mixed surfactant solution and 0.3 wt% ASB@PS can achieve an ultra-low IFT. The equivalent alkane carbon number (EACN) of Shengli crude oil varies between 9 and 12 [41]. This shows that ASB@PS matches the HLB with crude oil, satisfying the principle of “similar phase dissolution”. At the same time, it also matches the size of active components in crude oil.

**Figure 7:**
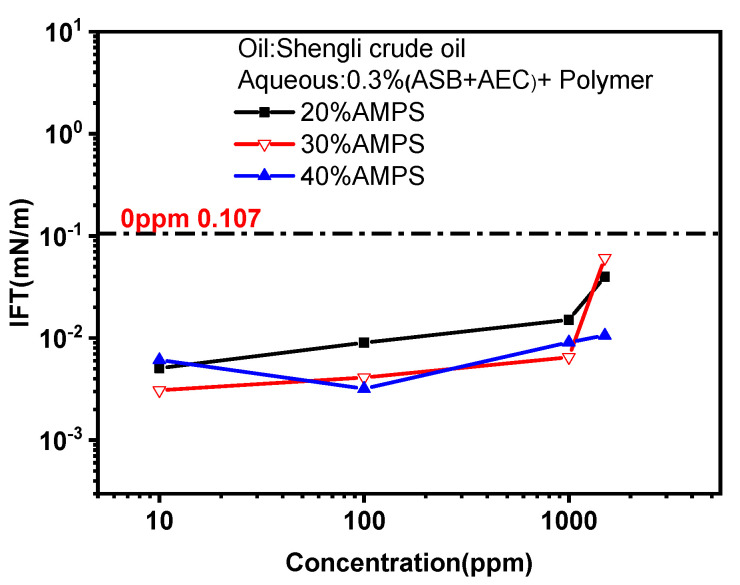
Effect of polymer concentration on equilibrium IFTs between 0.3 wt% ASB@AEC solution and Shengli crude oil.

As shown in Figure 7, the addition of different concentration polymers led to a significant decrease in the IFT between betaine ASB@AEC and crude oil, and an ultra-low IFT can be achieved at wide polymer concentration range. Moreover, the IFT slightly increases with the increase in polymer concentration. This may be due to the fact that the low polymer concentration may promote the interfacial film alignment and lead to the decrease in IFT, while the high concentration makes the interfacial alignment looser, and the IFT slightly increases [27,29]. The IFT between ASB@AEC and crude oil was only 10^−2^ mN/m when no polymer was added. The IFT between 0.3 wt% ASB@AEC solution and crude oil can be effectively reduced by adding polymer to achieve the ultra-low IFT. The n_min_ value of ASB@AEC is higher than 14, and the EACN of crude oil is lower than the EACN of crude oil. The addition of polymer decreases the n_min_ value of surfactant solution. Therefore, the mechanism of polymer action on the IFT between surfactant and crude oil is based on regulating the HLB, and the IFT decreases when the HLB improves.

**Figure 8:**
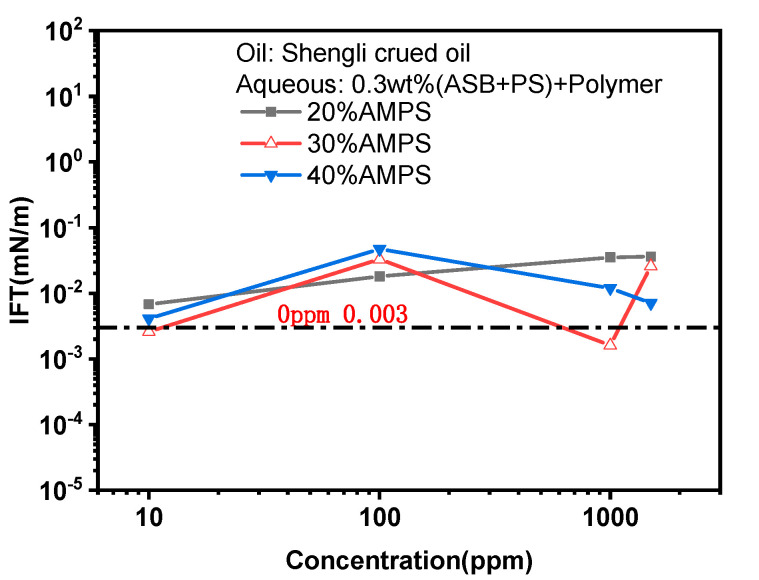
Effect of polymer concentration on equilibrium IFTs between 0.3 wt% ASB@PS solution and Shengli crude oil.

The effect of polymer concentration on equilibrium IFTs between 0.3 wt% ASB@PS solution and Shengli crude oil is plotted in Figure 8. Similar to the n-decane system, the IFT values increase with the addition of the polymers, which are quite different from those of ASB@AEC. This may be due to the fact that the EACN value of crude oil is generally around 10, and the addition of polymers reduces the n_min_ value of ASB@PS, destroying the HLB of the original system and resulting in an increase in the IFT.

## 3. Experimental Section

### 3.1. Materials

**Scheme 1:**
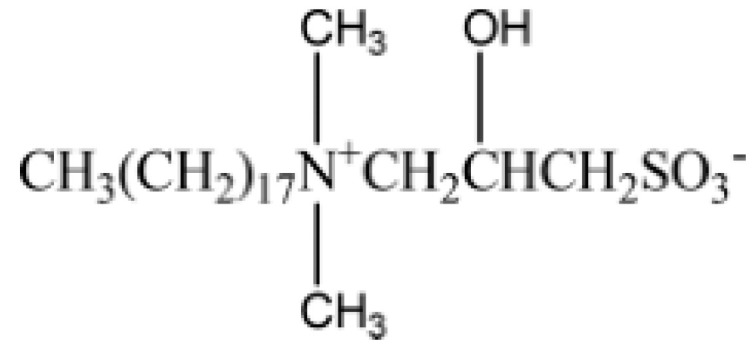
Structure of ASB structure.

The ASB@AEC (cetylsulfonbetaine + tetrainyl EO_3_ carboxylate) and ASB@PS (cetylsulfonbetaine + Shengli Petroleum sulfonate with an average molecular weight of 320) used in this study were provided by Shengli Oil Field (Dongying, Shandong, China). The ASB structure is shown in Scheme 1. Three kinds of polymers were obtained from Shengli Oil Field, China: low-hydrolysis new type III 1#, AMPS 20 wt%, hydrolysis degree 0, molecular weight 20.1 million; low hydrolyzed new type III 2#, AMPS 30 wt%, hydrolysis degree 0, molecular weight 19.5 million; low hydrolyzed new type III 3#, AMPS 40 wt%, hydrolysis degree 0, molecular weight 20.5 million. Alkane (>99 mol %) chain length C6–C14. The crude oil comes from Shengli Oil Field, and its density is 0.9157 g/cm^3^ at 80 °C. All inorganic reagents used (NaCl, NaHCO_3_, CaCl_2_, MgCl_2_) were of analytical grade, which purchased from Macklin Reagent Co., Shanghai, China. The simulated formation water was prepared with distilled water, whose composition is shown in Table 1. The pH values of simulated formation water, surfactant solution, and surfactant–polymer solution are shown in Table 2. The viscosity values of the surfactant–polymer solutions are shown in Table 3.

### 3.2. Apparatus and Methods

All IFT data in this paper were measured with a Texas-500C rotating droplet interfacial tensiometer (CNG Enterprises Ltd, POTOMAC, MD, USA). The surfactant solution was injected into the glass tube as the outer phase, and about 2 μL oil was injected into the middle of the tube as the inner phase. The volume ratio of oil to water was approximately 1:200. All experiments were operated at a rotating velocity of 5000 r/min. All experiments were carried out at 80.0 ± 0.5 °C. When the change of IFTs was less than 5% within 60 min, the values can be deemed to be the equilibrium IFTs. The measurement error of the IFT value is lower than 5%.

## 4. Conclusions

The dynamic IFTs of betaine ASB@AEC and ASB@PS solutions against n-alkanes and Shengli crude oil were studied in this paper. On the basis of the above work, the following conclusions can be drawn:

(1) The IFT between betaine ASB and n-alkanes can be reduced to an ultra-low value through compounding with an anionic surfactant PS and an extended anionic surfactant AEC, respectively. ASB@AEC is very oil-soluble, with an n_min_ value ≥14 and can produce ultra-low IFTs against oils C12–C14. On the other hand, ASB@PS is relatively water-soluble, with an n_min_ value of 10 and can achieve an ultra-low IFT against oils C8–C11.
(2) The water-solubility of both ASB@PS and ASB@AEC is enhanced by the addition of water-soluble polymers. The HLB of ASB@AEC solution becomes better when the oil is decane after the addition of polymers, and the IFT decreases as a result. On the contrary, the antagonistic effect can be observed for ASB@PS in reducing IFT against decane when polymers are added, which can be attributed to the deviation of the HLB.
(3) Similar to the IFT against decane, the addition of polymers decreases the nmin value of the surfactant solution and results in better and worse HLBs for ASB@AEC and ASB@PS against crude oil, respectively. Therefore, the IFTs of ASB@AEC solutions against crude oil decrease to ultra-low values therough the addition of polymers.

## Figures and Tables

**Table 1:** Composition analysis of formation brine.

Components	Cl^−^	SO_4_^2−^	CO_3_^2−^	HCO_3_^-^	Na^+^+K^+^	Ca^2+^	Mg^2+^	TDS
Concentration (mg/L)	17422	0	0	596	9665	1079	355	29117

**Table 2:** pH of the EOR surfactant solutions prepared by formation brine (25 °C).

Solutions	pH
Formation brine	7.78
0.3 wt%ASB@AEC	7.92
0.3 wt% ASB@PS	8.05
0.3 wt% ASB@AEC + 1500 ppm 20 wt%AMPS polymer	7.89
0.3 wt% ASB@PS + 1500 ppm 20 wt%AMPS polymer	8.03

**Table 3:** The viscosity (cP) of surfactant–polymer solutions (80 °C).

Polymer Concentration (ppm)	10	100	1000	1500
0.3 wt%ASB@AEC + 20 wt%AMPS polymer	0.9	1.1	4.1	6.9
0.3 wt%ASB@PS + 20 wt%AMPS polymer	0.8	1	3.8	6.7

## Sample Availability

Samples of the compounds ASB, AEC and PS are available from the authors.
